# 

**Published:** 1880-01

**Authors:** 


					﻿Whole Number,
CCCCXVI
January, 1880.
Number 1,
Vol. XL.
THE
CHICAGO
M E D I C A L
J ournal and Examiner
EDITORS:
JAS. NEVINS HYDE, A.M., M.D. N. S. DAVIS, M.D.rLL.D.
DANIEL R. BROWER, M.D.
CHICAGO:
PUBLISHED BY THE CHICAGO MEDIC AL P/iESS ASSOCIATION.
188 Clark Street.
tyRead the Notice of this Journal among Advertisements.
				

